# Attainable region analysis for continuous production of second generation bioethanol

**DOI:** 10.1186/1754-6834-6-171

**Published:** 2013-11-29

**Authors:** Felipe Scott, Raúl Conejeros, Germán Aroca

**Affiliations:** 1 School of Biochemical Engineering, Pontificia Universidad Católica de Valparaíso, Av. Brasil 2147, Valparaíso, Chile; 2 Bioenercel S.A. Barrio Universitario s/n, Ideaincuba building, Concepción, Chile

## Abstract

**Background:**

Despite its semi-commercial status, ethanol production from lignocellulosics presents many complexities not yet fully solved. Since the pretreatment stage has been recognized as a complex and yield-determining step, it has been extensively studied. However, economic success of the production process also requires optimization of the biochemical conversion stage. This work addresses the search of bioreactor configurations with improved residence times for continuous enzymatic saccharification and fermentation operations. Instead of analyzing each possible configuration through simulation, we apply graphical methods to optimize the residence time of reactor networks composed of steady-state reactors. Although this can be easily made for processes described by a single kinetic expression, reactions under analysis do not exhibit this feature. Hence, the attainable region method, able to handle multiple species and its reactions, was applied for continuous reactors. Additionally, the effects of the sugars contained in the pretreatment liquor over the enzymatic hydrolysis and simultaneous saccharification and fermentation (SSF) were assessed.

**Results:**

We obtained candidate attainable regions for separate enzymatic hydrolysis and fermentation (SHF) and SSF operations, both fed with pretreated corn stover. Results show that, despite the complexity of the reaction networks and underlying kinetics, the reactor networks that minimize the residence time can be constructed by using plug flow reactors and continuous stirred tank reactors. Regarding the effect of soluble solids in the feed stream to the reactor network, for SHF higher glucose concentration and yield are achieved for enzymatic hydrolysis with washed solids. Similarly, for SSF, higher yields and bioethanol titers are obtained using this substrate.

**Conclusions:**

In this work, we demonstrated the capabilities of the attainable region analysis as a tool to assess the optimal reactor network with minimum residence time applied to the SHF and SSF operations for lignocellulosic ethanol production. The methodology can be readily modified to evaluate other kinetic models of different substrates, enzymes and microorganisms when available. From the obtained results, the most suitable reactor configuration considering residence time and rheological aspects is a continuous stirred tank reactor followed by a plug flow reactor (both in SSF mode) using washed solids as substrate.

## Background

Production of bioethanol from sugar and starch rich feedstocks, such as sugar cane (sucrose) or starchy materials (corn, wheat, sorghum) is done using microorganisms such as *S. cerevisiae* or *Z. mobilis* in a fermentation process [1]. Since, bioethanol has to be recovered from the mixture of water (as reaction media), residual sugars and nutrients, it is convenient to increase the concentration of initial sugars (for batch fermentations) or feed concentration (for continuous processes) in order to raise the bioethanol titers. Thus reducing the energy consumption and operating and capital expenditures in the distillation operation [2,3]. However, microorganisms suffer from inhibition at both high sugar and bioethanol concentration [4]. For alleviating ethanol inhibition, batch bioreactors and plug flow bioreactors (PFR) are the best options because they do not present back-mixing, which effectively reduces their time-averaged product inhibition [5]. Traditionally, batch fermentation has been used in the bioethanol industry especially for small scale-facilities, and the Moiller-Boinot process (a fed batch process with cell recovery) has been extensively used in Brazil [6]. For modern bioethanol production plants, the working volume of bioreactors is on the order of thousands of cubic meter. As an example, a total of 20 bioreactors, with a working volume of 3000 *m*^3^ each, were constructed in the Shandong province, China in 2003 [1]. For such large facilities, batch bioreactors are unattractive because of the longer operational downtimes associated with mash adding, broth harvesting and facility cleaning [1]. Continuous PFR conditions are difficult to achieve in a fermentation process due to its extended residence time and gas production, which induce mixing. In fact, residence time can be as long as 48 to 72 hours to achieve an ethanol concentration of 10 to 12% [7]. Since a cascade of continuous stirred tank reactors (CSTR) also contributes reducing end-product inhibition, this strategy has been practiced in the bioethanol industry [8]. Generally, a train of four to six CSTR connected in series are preferred because such design presents an adequate trade-off between the glucose fermentation kinetics and the capital investments for tank manufacture [1]. This widely known use of a cascade of CSTRs as a way to minimize the residence time of the system is theoretically valid only for processes with a fixed overall reaction stoichiometry, and that can be described by a single kinetic expression. Although this may hold for ethanol fermentation kinetics [8], for enzymatic saccharification and simultaneous saccharification and fermentation operations in lignocellulosic ethanol production, the reaction network cannot be reduced to a single kinetic expression. Hence, the classic graphical methods for residence time optimization of continuous bioreactors are no longer applicable.

Bioethanol production from lignocellulosic substrates comprises a pretreatment of the feedstock to increase its reactivity to further enzymatic degradation [9]. These biocatalysts break the structure of cellulose and hemicellulose, producing sugar monomers and oligomers, which are subsequently fermented to bioethanol. Even at high solid concentration in the enzymatic hydrolysis step, glucose concentration at the beginning of the fermentation stage will not normally exceed 145 g/L, even considering full cellulose to glucose conversion of a pulp with 20% DW solid content with 65% of cellulose. This value is rather modest compared with first generation bioethanol production. Although, inhibition by ethanol or sugar concentrations is reduced in bioethanol production from lignocellulosics, the enzymatic hydrolysis process has its own inhibition effects. Glucose, cellobiose and xylose have been reported to inhibit the reaction rates of cellulolytic enzymes [10]. Considering that in conventional fermentation processes using sugar and starchy materials, the inhibition problems have been minimized using adequate reactor configuration, the following question naturally arises: which are the most advantageous reactor arrangements in the hydrolysis and fermentation areas for the production of bioethanol from lignocellulosic materials?

Since the conventional use of graphical methods for residence time minimization of a reactor network is no longer applicable to the system under study due to its high number of reactions, we focus on more general optimization methodologies. Optimization of reacting systems involves solving the following reactor network synthesis (RNS) problem as stated by Biegler et al. [11]: “*Given the reaction stoichiometry and rate laws, initial feeds, a desired objective, and system constraints, what is the optimal reactor network structure? In particular: (i) What is the flow pattern of this network? (ii) Where should mixing occur in this network? (iii) Where should heating and cooling be applied in this network?”* Question (i) addresses the mixing patterns of the reactors in the reactor network. In idealized reactors, two extremes exist: no axial dispersion inside the reactor (PFR) and full axial dispersion (CSTR) [5]. Question (ii) inquires about which reactors in the network should be fed with fresh feed (F) and which reactors should be fed with a mixture of intermediate product streams. Finally, (iii) refers to the heat supply or withdrawal in the network, e.g. to improve selectivity by increasing the rate of certain reactions over the rest of the reactions in the reaction network.

The problem of RNS can be addressed by an approach based in mathematical optimization of a reactor network superstructure or by graphical methods. Optimization based approaches start by proposing a reactor superstructure where all the possible reactors, mixing streams and heat streams are included. Then, optimal candidates are determined by searching in this superstructure. The first attempt using this strategy considered axial dispersion models and recycle PFRs [12] and the resulting candidate structures were found using nonlinear programming. Later, the concept of modeling the superstructure as a mixed integer nonlinear programming (MINLP) formulation was introduced [13]. Although this formulation allows a more natural modeling approach, the resulting optimization problems are generally non-convex and, therefore, it is difficult to obtain a global solution. In recent years, research in this area has been devoted to overcoming difficulties associated with the non-convexity of the optimization problems using global optimization techniques [14,15].

Graphical methods for RNS include the Attainable Region (AR) analysis. This method has originated from the work of Horn [16], who defined the AR as the set of *all possible* values of the outlet stream variables which can be reached by any possible (physically realizable) steady-state reactor system from a given feed stream using only the processes of *reaction and mixing*[17,18]. Horn [16] showed that once the AR is obtained, then an optimization problem with reactor output concentration as decision variables was essentially solved. The attainable region can be constructed for a given reaction network with *n* chemical compounds in an *n-* dimensional space. Its construction is supported by the application of proposition and theorems [17,19-22] that describe properties of the AR. Despite these powerful theoretical advances, there exist no sufficient conditions for the AR. Hence, the regions that are calculated applying the known necessary conditions are termed candidate attainable regions (AR^c^). For two and three dimensions, graphical constructive methods can be derived from these propositions and theorems, thus greatly facilitating its application. A detailed treatment of the methods used in this work is given in the Methods section. For the readers acquainted with the existing theory and results of the AR, this section can be skipped. However, we recommend consulting the details concerning the kinetic models used for the enzymatic hydrolysis and fermentation reaction networks.

In this work, we analyzed the process synthesis of the enzymatic hydrolysis and fermentation operations for bioethanol production, applying for the first time the concept of the Attainable Region to these systems. Two scenarios are analyzed: (i) conversion of washed pretreated material to bioethanol and (ii) production of bioethanol from the discharge stream of the pretreatment reactor (solids and reaction liquor), from this point on non-separated pretreated material (nSPM). In each scenario, production of bioethanol from pretreated material is performed in one of two alternative configurations: continuous separated saccharification and fermentation (cSHF) or continuous simultaneous saccharification and fermentation (cSSF). In cSHF mode, pretreated corn stover is continuously fed to an enzymatic hydrolysis system and the stream leaving this operation is discharged to a continuous fermentation system. In cSSF mode, pretreated corn stover is hydrolyzed and the released sugars fermented in the same reactor. The main purpose of this work is to establish the most appropriate configurations for these systems. Our interest in investigating the effect of reactor configurations when washed and nSPM are used was motivated by the work of Hodge et al. [10], regarding the effect of sugars and acids released during pretreatment over the enzymatic hydrolysis. We believe that, since an important inhibitory effect over the enzyme activity is caused by the sugars in the pretreatment liquor [10], appropriate reactor configurations may mitigate this problem.

## Results and discussion

### Attainable region candidate for cSHF

**Figure 1:**
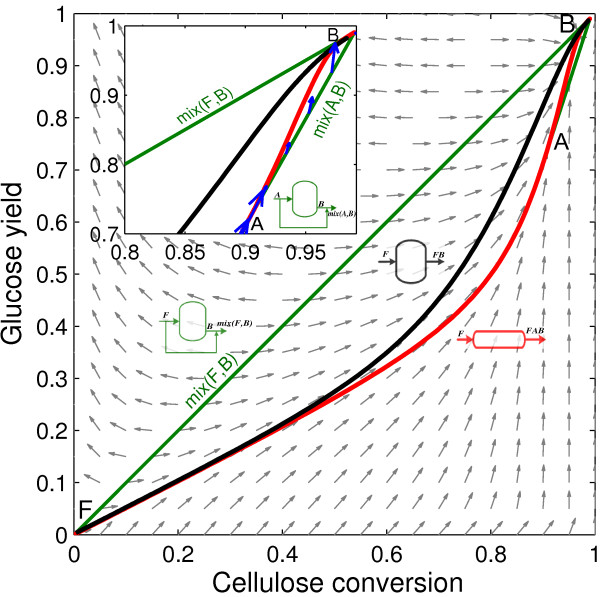
**Candidate attainable region for enzymatic hydrolysis in a bi-dimensional space of cellulose conversion and glucose yield.** Gray arrows correspond to the rate vector field, *r*(*c*). Blue arrows indicate the direction of the rate vector along the reactors trajectories. The AR^c^ is bounded by a PFR from F to A, a mixing line connecting points A and B to fill in the non-convex trajectory of the PFR and the mixing line connecting A and B. Every point inside this region is attainable using a suitable combination of reactors and mixing, but no point can be achieved outside it (in the complement of the AR^c^). Letters in italics above the fed streams to each reactor correspond to its composition, while the letters above the outlet streams denote all the composition produced for different residence times.

Four species take part in the enzymatic hydrolysis reaction: cellulose, glucose, cellobiose and water. Hence, it would be natural to describe the AR in a four-dimensional concentration space; however, species concentrations are not independent, and this allows calculating the changes in the number of moles in the enzymatic hydrolysis network as a function of cellulose and glucose molar changes (see the Dimensionality reduction techniques in the Methods section). We choose to display results in a dimensionless format using reaction conversions and yields (see Eq. (11) in the Methods section). In this two dimensional space (cellulose conversion and glucose yield), the enzymatic hydrolysis reaction network produces the AR^c^ shown in Figure 1 when the feed stream is composed of washed solids and a solid fraction of 0.2 is used. This corresponds to the minimum possible dimensionality of the AR^c^, in the following sections it will be expanded by incorporating the effect of the residence time.

Figure 1 will be used to illustrate the construction of a two dimensional AR^c^. Point F corresponds to feed stream composition, with zero glucose yield and cellulose conversion. To calculate the CSTR trajectory, the rate definition equations in Table 1 were substituted into Eq. (8), then the non-linear system of equations was solved for increasing residence time values until full conversion was achieved. This procedure is detailed in the Methods section; from this point on, we will refer to it as the calculation of a CSTR trajectory with a given feed composition. The PFR trajectory was calculated by integrating the system of differential equations obtained by substituting the enzymatic hydrolysis rate equations in Table 1 into Eq. (7). From now on, this procedure will be identified as the calculation of a PFR trajectory from a given point, which corresponds to its feed stream composition. Results show that the AR^c^ is bounded (below) by a PFR from feed point (F) up to point A. Figure 1 also shows the rate field, the rate vector evaluated for each point in concentration space. As it can be seen, the PFR trajectory is tangent to the rate field at every point along its path. Between point A and the equilibrium point B, the PFR trajectory is not convex and hence the AR^c^ is bounded by a by-pass reactor with a feed stream with the composition of point A (line *mix *(*A*, *B*) in Figure 1). This by-pass reactor can be either a CSTR or a PFR fed with a stream of composition A and operating with a residence time such that the composition of the outlet stream is B. To build the line connecting A and B, *mix*(*A*, *B*), the by-pass stream with composition A is mixed with the outlet stream of a PFR or CSTR with composition B according to the mixing equation, Eq. (10). The subplot in Figure 1 gives a detailed view of this section, indicating also that all the rate vectors along the AR^c^ boundary points inward or are tangent to the boundary and no rate vector outside the AR^c^, points inwards to the AR^c^ when reflected. As was proven by Glasser et al. [17], this indicates that the AR^c^ cannot be further extended by a PFR, a CSTR or mixing operations because all necessary conditions are met. The line connecting F and B corresponds to a bypass PFR or CSTR with feed composition equal to F. The derived AR^c^ satisfies all the necessary conditions listed for a two dimensional AR.

**Table 1:** Rate balance equations per compound for cSHF and cSSF operations

**Processes**	**Operations**	**Rate equations ( *r *** **( *c *) vector)**
cSHF	(1) Enzymatic saccharification	*r*_*S*_ = - *r*_1_ - *r*_2_
		*r*_*B*_ = *r*_1_ - *r*_2_
		*r*_*G*_ = *r*_2_ + *r*_3_
	(2) Ethanol fermentation	$r_{x}=r_{x}^{F}$
		$$r_{G}=r_{G}^{F}$$
		$$r_{P}=r_{P}^{F}$$
cSSF	1 and 2	*r*_*S*_ = - *r*_1_ - *r*_2_
		*r*_*B*_ = *r*_1_ - *r*_2_
		$$r_{G}=r_{2}+r_{3}-r_{G}^{F}$$
		$$r_{x}=r_{x}^{F}$$
		$$r_{P}=r_{P}^{F}$$

Reaction rates for enzymatic saccharification are from Kadam et al. [24]. For glucose fermentation reaction rate definitions are presented in the text. Process cSSF combines both reaction rates through glucose as a common intermediate.

**Figure 2:**
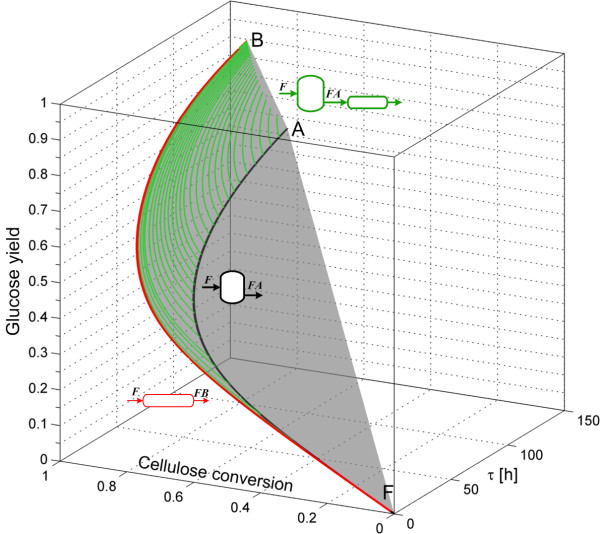
**Step 1 in AR**^**c **^**construction for enzymatic hydrolysis.** PFR and CSTR from feed point F, PFR with feed points over the CSTR trajectory and the convex hull of these trajectories (gray shaded region). The AR^c^ feed stream is washed solids at 0.2 solid fraction. Letters in italics above the fed streams to each reactor correspond to its composition, while the letters above the outlet streams denote all the composition produced for different residence times.

**Figure 3:**
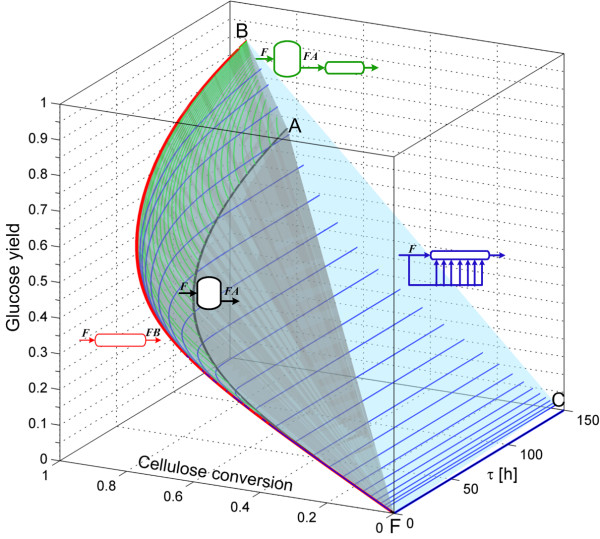
**Step 2 in AR**^**c **^**construction for enzymatic hydrolysis.** The AR^c^ is enlarged when DSR reactors are included. DSRs are calculated with constant feed rates, glucose yield and cellulose conversion decrease with larger feed rates. The AR^c^ feed stream is washed solids at 0.2 solid fraction and all reactors are fed with this stream as indicated by letters in italics above the feed streams.

Since, the two dimensional AR^c^ for enzymatic hydrolysis does not provide information about the residence time of the reactors, and as this parameter is related to the reactor capital cost, we constructed the AR^c^ in a three dimensional space of residence time, cellulose conversion and glucose yield. The stepwise procedure to construct the AR^c^ in this space is depicted in Figures 2 and 3. The first step is shown in Figure 2. From feed point F, the PFR trajectory $\bar{\left(\text{FB}\right)}$ is calculated up to a residence time of 150 h. Then the CSTR trajectory with feed composition F is calculated and the convex hull of both trajectories is computed. It is clear that the PFR trajectory is extreme, while the CSTR trajectory $\bar{\left(\text{FA}\right)}$ it is not since it is within the convex hull (shaded gray volume). It is possible to connect the PFR and CSTR trajectories using PFRs with feed points along the CSTR trajectory. These trajectories play an important role from a practical point of view as it will be discussed later. The next step is to calculate a set of constant *α* values DSRs (Figure 3), and the extreme DSR reactor (connecting the points F and C). These reactors further extend the AR^c^ from the situation shown in Figure 2, and the extreme DSR is completely built from a collection of extreme points (they lie in the boundary of the AR^c^ and not in its interior, see definition and notation in the Methods section). However, this reactor is of little practical significance since along its trajectory, almost no conversion of cellulose is obtained. This is due to a very high side-feed rate. The shaded region in Figure 2 is almost entirely contained in the convex hull formed by the constant DSRs and the extreme DSR (light blue shaded region in Figure 3), with the exception of the points along the PFR trajectory.

**Figure 4:**
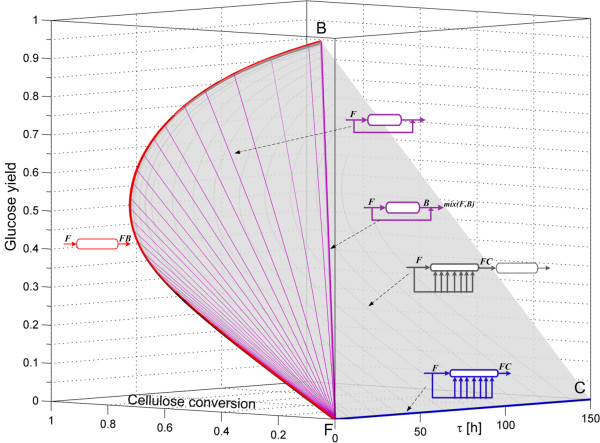
**AR**^**c **^**for enzymatic hydrolysis.** The ARc is made of three zones: the plane FBCF, made of mixing lines connecting point B and the extreme DSR line $\overset{¯}{\mathrm{FC}}$; the mixing lines connecting point F and points along the PFR trajectory (in magenta); and, in the back of the figure, by PFR with feed composition along the extreme DSR line $\overset{¯}{\mathrm{FC}}$.

Finally, the complete AR^c^ for the enzymatic hydrolysis reaction network is shown in Figure 4. Its boundary is formed by the PFR trajectory $\bar{\left(\text{FB}\right)}$, the PFR bypass reactor with feed point at F, the plane FBCF formed by PFR bypass reactors with feed along the extreme DSR trajectory, and finally by the trajectories in the *back* of the AR^c^ which correspond to PFRs with feed points along the extreme DSR trajectory.

**Figure 5:**
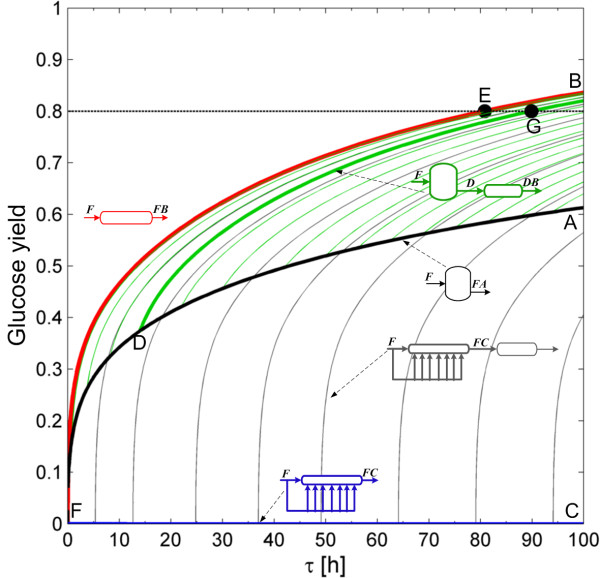
**Projection of the AR**^**c **^**in the residence time and glucose yield space.** For every glucose yield, the smallest residence time reactor is a PFR, but a reactor network composed of a CSTR followed by a PFR requires similar residence time to achieve identical glucose yields (as in point G). Letters above the feed and outlet streams denote its concentration.

**Figure 6:**
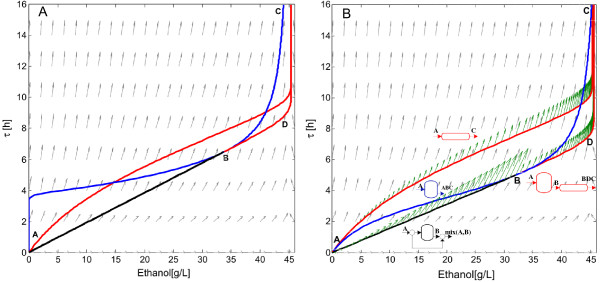
**Candidate attainable region for ethanol production using *S. cerevisiae.*** Left **(A)**, the feed stream to the CSTR does not contain cells and right **(B)** the feed stream to the CSTR contains 1 g/L of *S. cerevisiae*. In both cases, the feed stream to the PFR reactor contains 1 g/L of cells and 100 g/L of glucose. Gray arrows correspond to the rate vector field, *r*(*c*), green arrows indicate the direction of the rate vector along the reactors trajectories.

Figure 5 shows the residence time required to achieve different glucose yields. This is a projection of the three dimensional AR^c^ into a two dimensional space of residence time and glucose yield. A PFR reactor bounds the AR^c^, and this reactor represents the *lowest* residence time reactor for any glucose yield. For example, if a 0.8 glucose yield has to be attained, then the reactor with the smallest residence time is a PFR (point E in Figure 6 with *τ* = 80.85 *h*), followed by the reactor configurations constituted by a PFR with feed point along the CSTR, from now on CSTR → PFR, (such as point G in Figure 6 with *τ* = 89.90 *h*). A very particular reactor configuration also plays a role in this discussion as evidenced by the gray lines in Figure 5. These reactor configurations correspond to a PFR reactor with feed point along the extreme DSR (linen $\overset{¯}{\mathrm{FC}}$ in blue) in Figures 4 and 5. Although they have similar residence time and glucose yield as the CSTR → PFR configuration, the extra complexity of feeding a solid substrate along the DSR trajectory, makes PFR and CSTR → PFR configurations preferable. In fact, although a PFR has the smallest residence time, from an operative viewpoint it does not represent the best configuration. Because solids are involved in the reaction, it will be difficult to achieve a real plug-flow behavior. Furthermore, since at high solid fractions the pulp -water mixture has extremely high viscosities and yield stress [23], a CSTR → PFR it is a better configuration since the solid fraction inside the CSTR corresponds to the solid fraction in its outlet stream. This allows having a feed stream that behaves as high viscosity mixture while the reactor content behaves as a pumpable liquid. Literature evidence shows that starting at 20% total solids, the pretreated biomass behaves as a pourable liquid (at a yield stress below 10 Pa) for cellulose conversions greater than 40% [24]. The point marked D in Figure 5 corresponds to a residence time of 14 h over the CSTR trajectory, a glucose yield of 0.377 and a cellulose conversion of 0.627, hence at this points it is expected that the reaction mixture behaves as a pourable liquid, thus facilitating its flow to a PFR reactor and reducing the mixing energy requirements, since as it is a CSTR the outlet stream has the same properties as the reactor content.

### Attainable region candidate for glucose fermentation

Glucose fermentation must follow enzymatic hydrolysis in the cSHF operation. Figure 6 shows the candidate AR for bioethanol production using *S. cerevisiae* and the effect of cell feeding to the CSTR reactor. The feed stream to the PFR should always contain cells because cell growth is an autocatalytic reaction; in Figure 6B, cell concentration corresponds to 1 g/L. When no cells are supplied to a CSTR in the feed stream, no ethanol production occurs until residence time reaches 4 h. Before this residence time, the feed rate exceeds the growth rate of the cells and the culture is washed out from the fermentor.

From feed point A to the point marked B, the CSTR trajectory describes a non-convex curve, so a mixing line connecting the feed composition to point B (line $\overset{¯}{\mathrm{AB}}$) can be used to extend the AR. Point B coincides with the point on the curve of the CSTR where the rate vector starts pointing outside the AR. Thus, at point B the AR^c^ can be extended by a PFR with feed concentrations in B. The line $\overset{¯}{\mathrm{AB}}$ and the CSTR followed by PFR trajectory define the boundary of the attainable region. Along this boundary lies the *minimum residence time reactor configurations* for a given bioethanol concentration (or yield).

### Attainable region candidate for cSSF

**Figure 7:**
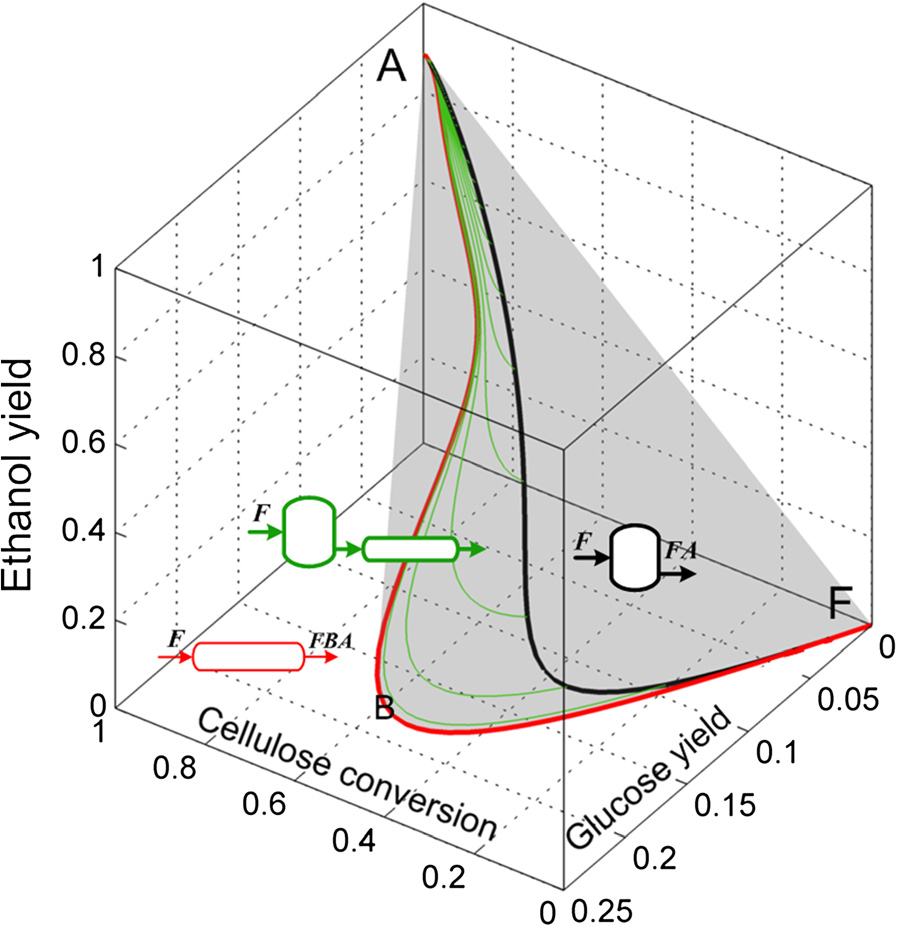
**First step in the AR**^**c **^**construction for cSSF.** PFR and CSTR from feed point F to point A (full ethanol yield). Green trajectories correspond to PFR with feed points along the CSTR. The gray shaded region represents the convex hull of all the trajectories. The feed stream corresponds to washed solids at 0.2 solid fraction and all reactors are fed with F as denoted by italic letters above the reactor’s feeds.

Accordingly to the analysis presented in the Methods section, the changes in the number of moles in the cSSF reaction network can be expressed as a function of the changes in the number of moles of cellulose, glucose and ethanol. We start the AR^c^ construction for the cSSF system by drawing the CSTR trajectory from the feed-point (F) as well as the PFR from this point, the CSTR → PFR trajectories and the convex hull of this region (Figure 7), the algorithmic procedure used for the construction of the AR^c^ for cSSF is presented in the Additional file 1. Up to this point, the extreme points are F (feed point), A (the equilibrium point of complete cellulose conversion) and all the points on the PFR trajectory with F as feed composition. The CSTR trajectory lies within the convex hull, and hence no extreme CSTR exists in this system (with the exception of points F and A of course).

**Figure 8:**
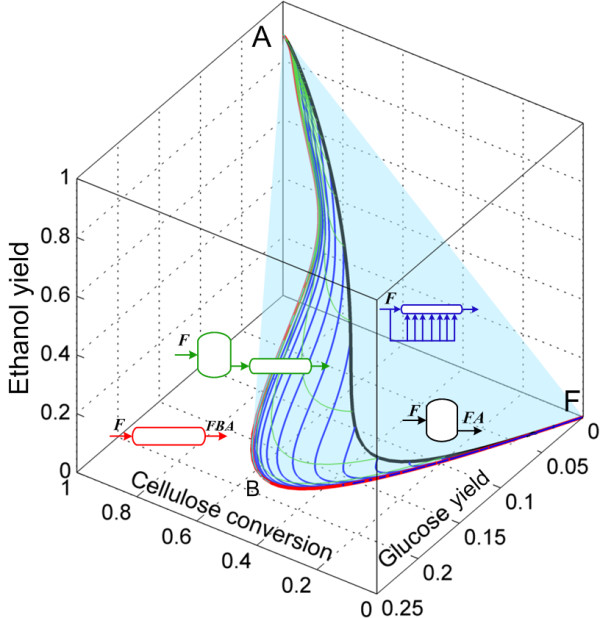
**Step 2 in the AR**^**c **^**construction for cSSF.** Addition of constant fed policy DSRs trajectories with F as feed composition (no ethanol or glucose) and side-feed composition equal to F. These trajectories do not enlarge the AR^c^ from the situation shown in Figure 7.

**Figure 9:**
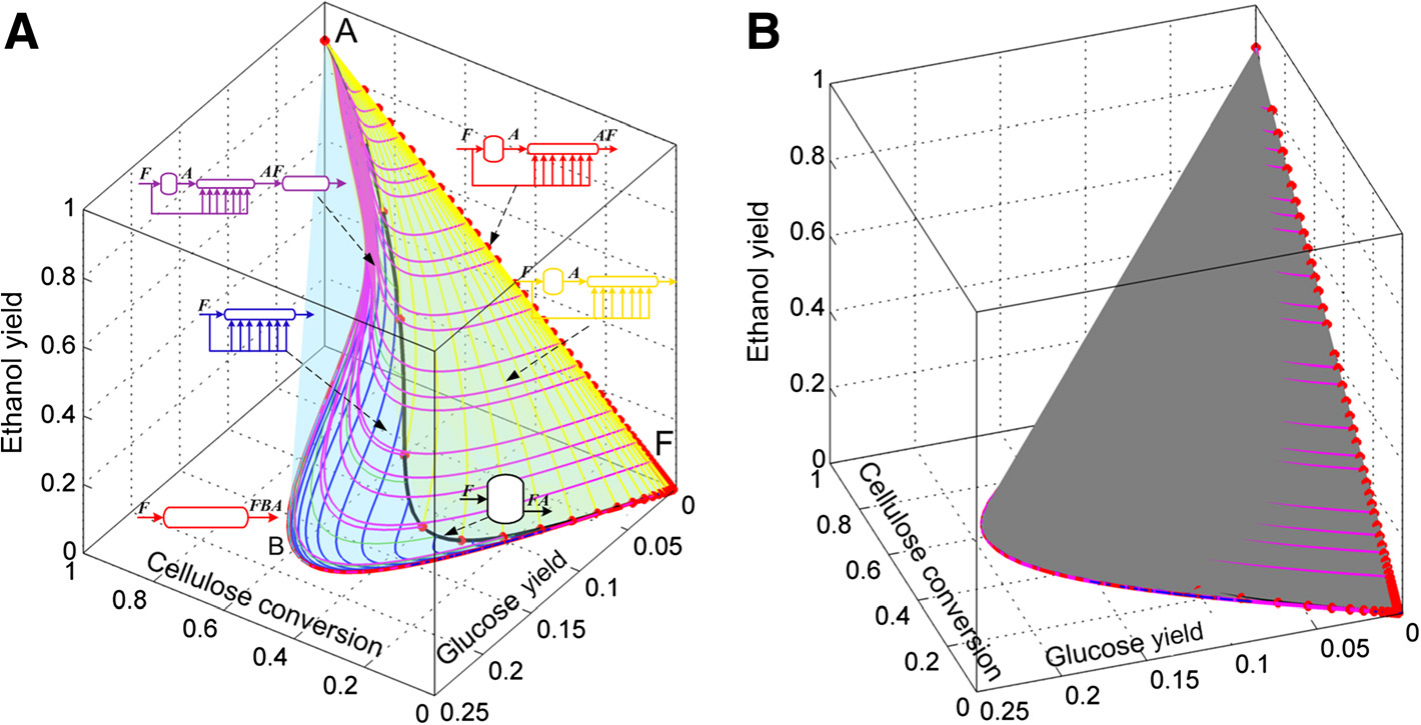
**Step 3 in the AR**^**c **^**construction for cSSF.** Constant fed policy DSRs from A (feed composition) and with side-feed composition equal to F. These DSRs enlarge the AR^c^ from the situation shown in Figure 8. Left **(A)** a transparent view of the convex hull showing its interior and right **(B)** the convex hull was shaded gray.

Figure 8 shows constant feeding policy DSR trajectories starting from F. As *α* values (see Eq. (9)) increase from 0 to 500 *m*^*3*^*/h*, the trajectories of the DSRs bend and do not reach the point A, but they intersect the CSTR trajectory. This implies that no extreme DSR trajectory from F exists, and hence the AR^c^ is not expanded by these reactors. When the trajectories of the constant *α* DSRs from point A are included (Figure 9) these form an extreme DSR path (red points along the AF line) and the PFRs with fed point along the extreme DSR trajectory (exDSR → PFR) form new extreme points. However, the newly included exDSR → PFR are not extreme for every residence time along their trajectories, in fact as it can be seen in Figure 9B all the exDSR → PFR start at the extreme DSR points and after some residence time they *dive* into the convex hull. At each of the final points of these exDSR → PFR trajectories (the points where the trajectories dive into the convex hull), a bypass reactor connecting point A and these points exists. Although these exDSR → PFR are important as they constitute part of the AR^c^ boundary, they have little practical value for two reasons. Firstly, they originate along the extreme DSR trajectory starting on point A, this means that they start at a very high residence time, and they further extend it. Secondly, along its trajectory reactions produce glucose but almost no bioethanol until a very high residence time (or cellulose conversions in Figure 9A).

**Figure 10:**
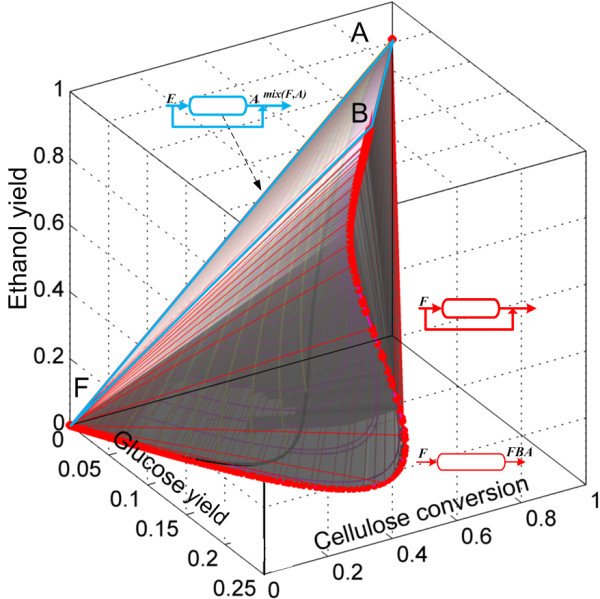
**AR**^**c **^**for continuous saccharification and fermentation of pretreated corn stover.** PFR from feed point F is extreme up to point B. The rest of the AR is composed of mixing lines, except by the lines shown in magenta in Figure 9B (it is not possible to see these lines in the view shown in Figure 10).

Finally, the complete AR^c^ is shown in Figure 10. In this view of the AR^c^, the extreme points along the PFR (which are also extreme points for the DSR from F) are shown as red dots in the trajectory $\overset{¯}{\mathrm{FB}}$. In point B, the PFR trajectory is no longer extreme since a mixing line connecting points A and B can be used to complete the convex hull, this creates a plane (AFBA) made of by-pass reactors.

**Figure 11:**
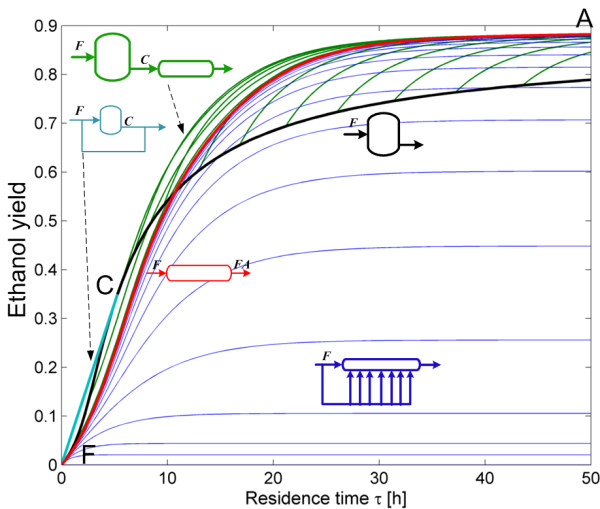
**Residence time for the reactors in the AR**^**c **^**for cSSF.** Projection in the ethanol yield and residence time space. The minimum residence time reactor network, for ethanol yields above 0.35, is composed of a CSTR reactor with feed composition F followed by a PFR reactor.

As residence time is of great importance from a cost engineering point of view, the projection of the AR^c^ into a residence time and bioethanol yield plane is presented in Figure 11. As it can be seen, constant *α* DSRs do not play a relevant role (particularly for large values of *α* since at the same residence time, yield decreases with increments in *α*) as they produce small ethanol yields even at elevated residence times. The minimal residence time reactor configuration change as the residence time or yield progresses. From F to C, the minimum residence time configuration is a by-pass CSTR connecting point F and C. This is so, because for any given ethanol yield between 0 and 0.35, a horizontal line $\left(l\right)$ extended from the yield value in the ordinate intersects the by-pass reactor trajectory in the first place. Although intersections of l and other reactors for higher residence times are possible, they are neither relevant nor convenient. For yields greater than 0.35, the minimal residence time configurations is represented by a PFR with feed point in C. This is a remarkable result as it suggests that a very simple reactor arrangement (CSTR → PFR) can be used as the minimal residence time configuration. In addition, as it was discussed for the minimal residence time configurations for cSHF, the CSTR → PFR arrangement is of practical value since allows taking advantage of a CSTR’s property: the reactor always operates at the outlet conditions and not in the feed conditions. This results in and operation with a pourable liquid instead of a viscous solid/liquid mixture.

### Comparison of cSSH and cSHF operations with washed solids and non-separated pretreated material

**Figure 12:**
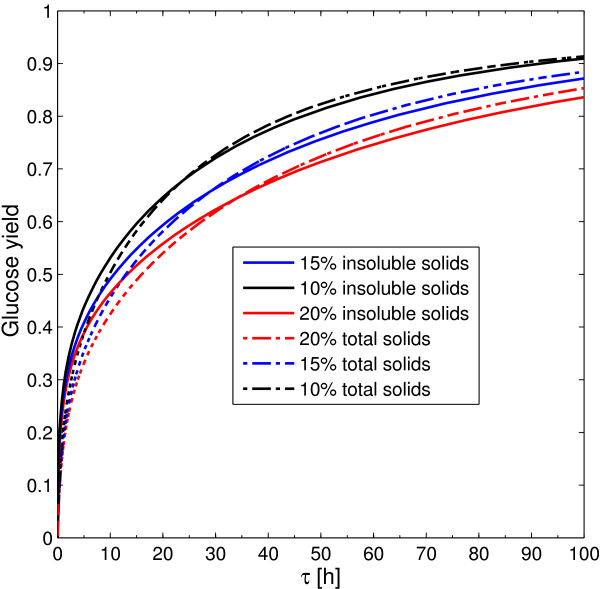
**AR**^**c **^**for cSHF at different solid loading and feed composition.** Effect of solid loading on continuous enzymatic hydrolysis and comparison of the operation with washed solids (solid lines) and non-separated pretreated material (dashed lines).

For enzymatic hydrolysis, the boundary of the AR^c^ is invariably specified by a PFR reactor, despite the feed point F corresponds to washed solids or non-separated pretreated material. Similarly, the solid fraction does not change this situation. Although Figure 12 shows higher glucose yields for cSHF operation with non-separated pretreated material, this does not imply a higher glucose concentration. In fact, when non-separated pretreated material is used, an important fraction of the soluble solids corresponds to xylose. This implies that, at equal total solid and insoluble solid fractions there is more potentially obtainable glucose for washed solids. With potentially obtainable glucose, we refer to the glucose that would be obtained if all the cellulose could be converted to glucose in an enzymatic hydrolysis process.

When washed solids and non-separated pretreated material operations are compared in a common potentially obtainable glucose basis (15% solid fraction for washed solids and 20% for non-separated pretreated material), cellulose conversion is higher for washed solids as it is shown in Figure 12.

When glucose yields at 100 h, for washed solids and nSPM, are plotted against the solid content, then negative slope *straight lines* are obtained with correlation coefficients of 0.9998 and 0.9996 for washed solids and non-separated pretreated material respectively. This behavior was already observed for both SSF and enzymatic hydrolysis along several experimental data sets independently published by several authors and analyzed by Kristensen et al. [25]. It is interesting to point out that we are using a kinetic model published in 2004, and the observation of Kristensen et al. [25] was made on 2009, this means that with an appropriate simulation effort, this conclusion could have been drawn from *in silico* analysis several years earlier.

**Figure 13:**
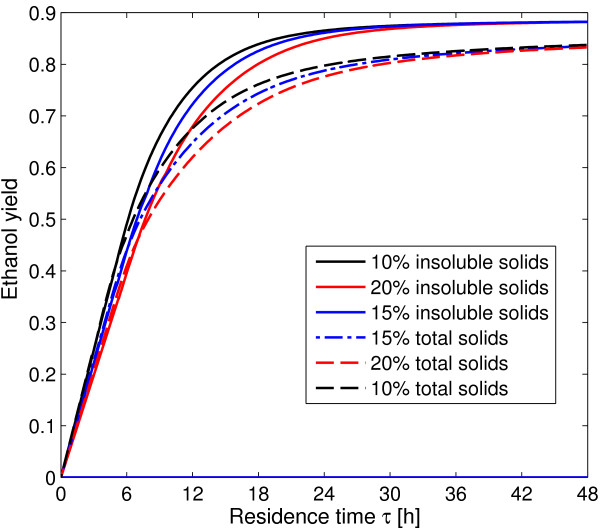
**AR**^**c **^**for cSSF at different solid loading and feed composition.** Effect of solid loading on cSSF and comparison of cSSF operation with washed (solid lines) and non-separated pretreated material (dashed lines).

The effect of the solid loading over cSSF operation and the effect of cSSF operation with washed solids or non-separated material is shown in Figure 13. It is very interesting to note that, as opposed to enzymatic hydrolysis (Figure 12), at short times all the solid fractions results in the same bioethanol yield. This result opposes to the linear decrease reported by Kristensen et al. [25] for different SSF experimental sets. The effect of operation with non-separated solids is far more harmful on cSSF compared to enzymatic hydrolysis. Figure 13 shows that when non-separated pretreated material is used, bioethanol yield decreases by nearly 5% at 48 h residence time. This effect can only be surpassed when the initial xylose fraction in the feed is taken as zero (instead of 0.279) indicating that the model predicts a strong inhibitory effect of this sugar over the enzymatic conversion of cellulose.

Results suggest that non-separated pretreated material should only be used if a xylose co-fermenting microorganism is available; otherwise, the strong inhibitory effect exerted by xylose over the cellulolytic enzymes causes an important reduction of cellulose conversion, and hence in the amount of bioethanol obtained from the cellulosic fraction of the pretreated material.

### Validity of the results

Results presented so far suggest that a CSTR followed by a PFR has the minimal residence time for cSSF and bioethanol production, and a near minimal residence time for cSHF. Furthermore, this design entails significant benefits from a rheological point of view. However, our results were obtained with two among the many available reaction kinetics for the processes under analysis. Hence, we do not claim that the suggested reactor configuration will be the optimal case for any reaction network and kinetic expressions in the cSHF and cSSF systems. However, literature evidence supports that for auto-catalytic reactions and product-inhibited bio-reaction networks, a combination of CSTR followed by PFR or a series of CSTRs often have the minimal residence time despite its particular kinetic parameter values [8,26] for a reaction network that can be expressed as a single reaction kinetic.

From a practical point of view, the PFR operation it is not technically possible because of the gas production in the fermentation, thus a series of CSTR can be used to mimic this reactor.

## Conclusions

An attainable region analysis was performed over the conversion of pretreated corn stover to bioethanol, considering two processes: SHF and SSF and washed and non-washed material. Independent kinetic models were used for each operation, i.e.: enzymatic saccharification, fermentation, and simultaneous saccharification and fermentation, in continuous operation. Our aim was to identify the reactor network configurations that provide lower residence times for both processes. Due to the high number of chemical species involved in the reaction network, and hence the high dimensionality of the AR, it was expected that the by-pass and/or DSR would shape the boundaries of the AR for minimum residence time, however these are not involved in the configurations that resulted in the lowest residence time.

For SHF, the saccharification reaction must be performed in a PFR to achieve the minimum residence time; however because it is unfeasible from a technical point of view due to the rheological restrictions of the system, the most adequate configuration with technical feasibility and with the closest residence time to the optimum is a CSTR followed by a PFR. For the fermentation operation, the minimum residence time is achieved in a reactor configuration of a CSTR followed by a PFR.

For SSF, the minimum residence time was obtained using a CSTR followed by a PFR, being the enzymatic saccharification and fermentation reactions carried out simultaneously in both reactors at isothermal conditions.

Regarding the effect of soluble solids in the reactor network feed stream; for cSHF, higher glucose concentration and yield are achieved for enzymatic hydrolysis with washed solids compared with non-separated pretreated material. For cSSF, higher yields and bioethanol titers were obtained when using washed solids.

In this work, we demonstrated the capabilities of the attainable region analysis as a tool to assess the optimal reactor network with minimum residence time applied to the SHF and SSF operations for lignocellulosic ethanol production. According to the kinetic models used in this study, the most appropriate reactor configuration for ethanol production from pretreated corn stover is a CSTR followed by a PFR, both operating in cSSF mode, and with washed pretreated material as substrate. The methodology can be readily modified to evaluate other kinetic models of different substrates, enzymes and microorganisms when available.

## Methods

All the methodology described in this section is oriented to construct the AR^c^ for the different scenarios described in the Background section. cSHF and cSSF AR^c^s were constructed for washed solids and nSPM. Unless otherwise specified, the solid fraction is equal to 0.2 total dried solids. For enzymatic hydrolysis simulation the temperature was taken as 50°C, and for cSSF and fermentations temperature is 32°C. In both cSHF and cSSF operations, enzyme doses were established as 45 mg protein/g cellulose (CPN commercial cellulase, Iogen Corp., Ottawa, Ontario, Canada) [27].

### Pretreated material

The pretreated material was assumed to be corn stover pretreated using dilute acid hydrolysis. The material composition was adapted from NREL’s 2011 report on biochemical conversion of corn stover to ethanol [28]. Only compounds taking part in the kinetic models used in this study were considered for calculations, with this consideration the soluble and insoluble compositions in the pretreated corn stover are given as follow (DW%): cellulose, 44.3; xylose, 27.9; lignin, 21.1; glucose, 6.0 and xylan, 0.7. Considering these compounds only, the total solid (soluble and insoluble) fraction is 0.148, the rest being water. When washed solids are used, the solid fraction is assumed to be composed only of cellulose, lignin and xylan. Subtracting the soluble solids from the composition given in NREL’s 2011 report [28], the washed solid is composed of (DW%): cellulose, 67.0; lignin, 32.0 and xylan, 1.1.

### Reaction kinetics

Enzymatic hydrolysis reactions scheme considers cellulose hydrolysis to cellobiose, Eq. (1) and rate *r*_1_, catalyzed by *endo-β -*1,4-glucanase [EC 3.2.1.4] and *exo*- *β*-1,4 cellobiohydrolase [EC 3.2.1.91]; cellobiose hydrolysis to glucose, Eq. (2) and rate *r*_2_, by *β* - glucosidase [EC 3.2.1.21] and direct cellulose to glucose hydrolysis, Eq. (3) and rate *r*_3_, by *exo*-*β*-1,4 cellobiohydrolase [EC 3.2.1.91] and *exo*-*β*-1,4 glycohydrolase [EC 3.2.1.74] [27]. The reaction network can be summarized as in Eqs. (1) to (3), and the kinetic expressions for reaction rates were taken from Kadam et al. [27]. The kinetic expressions are temperature dependent, and consider inhibitory effects of the sugars released from cellulose over the enzymes activity. Furthermore, the model incorporates an inhibitory effect of xylose concentration.

(1) $${\left(C_{6}H_{10}O_{5}\right)}_{n}+H_{2}O\overset{r_{1}}{\to }C_{12}H_{22}O_{11}+{\left(C_{6}H_{10}O_{5}\right)}_{n-2}$$

(2) $$C_{12}H_{22}O_{11}+H_{2}O\overset{r_{2}}{\to }2C_{6}H_{12}O_{6}$$

(3) $${\left(C_{6}H_{10}O_{5}\right)}_{n}+H_{2}O\overset{r_{3}}{\to }C_{6}H_{12}O_{6}+{\left(C_{6}H_{10}O_{5}\right)}_{n-1}$$

The analysis of the fermentation reaction network is based in the model presented by Rivera et al*.*[29]. The model involves the production of ethanol and *S. cerevisiae* considering biomass growth rate inhibition by substrate, ethanol and biomass concentrations. The kinetic expressions are reproduced in Eq. (4) as they will play a role in the analysis of the AR for continuous fermentation and cSSF.

(4) $$\begin{array}{l} \mu =\frac{\mu_{\max}G}{K_{G}+G}e^{-K_{i}G}{\left(1-\frac{X}{X_{\max}}\right)}^{m}{\left(1-\frac{P}{P_{\max}}\right)}^{n}\\ r_{x}^{F}=\mathrm{\mu X}\\ r_{G}^{F}=-\left(\frac{r_{x}}{Y_{x}}+m_{s}\right)X\\ r_{P}^{F}=\left(Y_{P}r_{x}+m_{p}\right)X \end{array}$$

In Eq. (4) G, X and P correspond to glucose, biomass and ethanol concentration respectively. In Eq. (4), *μ*_*max*_, *P*_*max*_, *X*_*max*_, *Y*_*x*_ and *Y*_*P*_ are functions of the fermentation temperature. Details regarding these expressions and the values of the constants in the model can be found elsewhere [29]. The above defined reaction rates describe the reaction processes that participate in the cSHF and cSSF operations. The particular reaction rates for each component in cSHF and cSSF processes are shown in Table 1.

We consider that the non-separated pretreated material is free of fermentation inhibitors, because they were not produced due to optimized pretreatment conditions, or they were removed using suitable technologies. This allows us to concentrate our attention on the inhibitory effects of sugars over the enzymatic reaction rates as these compounds cannot be removed unless washed substrate is used. Additionally, the kinetic models used do not incorporate the effect of the inhibitors such as furfural or acetic acid. If, under these considerations the operation with non-separated pretreated material results in worst results compared to washed material, then this simplification will not be important.

### Attainable region: definitions and notation

This section introduces the definitions required to understand the fundamental aspects of the attainable region analysis. Let us begin by assuming that a concentration vector exists in *R*^*n*^ (with *n* the number of reacting species) for the reaction network under analysis, this *concentration vector* ***c*** (Eq. 5) is formed by the molar (or mass) concentrations of the *n* reacting species and by the residence time of the reactor. Hence, ***c*** represents the instantaneous concentration within a reactor. For a given value of the concentration vector (***c***), it is possible to write the rate of formation of each species as the *rate vector* ***r*** (***c***). Note that the rate vector can be computed at any point in the concentration space and thus a vector field in *R*^*n*^ can be calculated, the *rate field.* This field will play an important role when defining the idealized reactors, since the equations that define them constrain which concentrations can be achieved, creating trajectories in the concentration space that are tightly connected with the rate field.

(5) $$c=\left\{c_{1},c_{2},\ldots ,c_{n},\tau \right\}$$

(6) $$r=\left\{r_{1},r_{2},\ldots ,r_{n},\tau \right\}$$

As it was stated in the definition of the AR given earlier, mixing and reaction are the two operations that allow reaching all the points in the attainable region [17]. Furthermore, it was shown that only three idealized reactors, along with mixing between their input and output streams, are required to construct the AR [20]. These reactors are: the plug flow reactor (PFR), the continuous stirred tank reactor (CSTR) and the differential sidestream reactor (DSR). Their trajectories can be investigated by analyzing the equations that define its behavior (under constant density and isothermal operations).

(7) $$\frac{\mathrm{dc}}{\mathrm{d\tau}}=r\left(c\right),c\left(\tau =0\right)=c_{o}$$

Eq. (7) defines the PFR reactor trajectory in the concentration space as a function of its residence time (*τ*). From Eq. (7) it is evident that the concentrations mapped out by integrating the PFR equations produce a trajectory that is tangent to the rate vector at every point along the reactor’s path. On the other hand, a CSTR is defined by Eq. (8). Whereas PFR trajectories are calculated by integration, the trajectory associated with a CSTR is found by solving a system of nonlinear equations for a given value of residence time. For a particular value of *τ*, the CSTR has the property such that the vector defined by the difference between the outlet and feed concentrations $\left(c-c_{o}\right)$ is collinear with the rate vector.

(8) $$\;c-c_{o}=r\left(c\right)\tau$$

For two dimensional systems, the AR is constructed using only CSTRs and PFRs. However, in three or more dimensions differential sidestream reactors (DSR) play a role in shaping the AR boundary, DSRs are defined by Eq. (9).

(9) $$\frac{dc}{\mathrm{d\tau}}=r\left(c\right)+\alpha \left(c_{o}-c\right)c\left(\tau =0\right)=c_{o}$$

Physically, a DSR corresponds to a PFR with a side feed stream distributed all along its length. It is interesting to note that, if *α* is equal to zero, then we have a PFR and if *α* is equal to 1/*τ* and the reactor operates in stationary state, then the reactor behaves as a CSTR.

The particular combination of reactor types and their arrangement is called a reactor structure or reactor network. The operation of mixing applies over the outlet streams of reactors in the network, and over any given combination of points already attained in the AR (but not outside it, i.e. in the complement of the AR). When two streams with compositions **c**^1^ and **c**^2^ are mixed, at constant density, the compositions lie in the straight line between **c**^1^ and **c**^2^, Eq. (10).

(10) $$c=\gamma c^{1}+\left(1-\gamma \right)c^{2}$$

**Figure 14:**
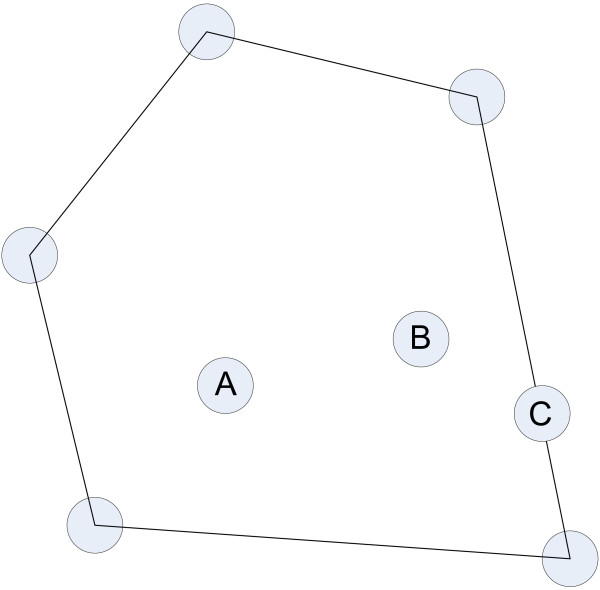
**Convex hull of a convex set S in *R***^**2**^**. **The convex hull of the points is shown. Points in the vertices are extreme points, but points A, B and C are not.

With *γ* a real number in the range [0,1]. This is usually referred to as the lever-arm rule, and can be derived from mass balance equations. To clarify the mixing operation concept, consider two streams 1 and 2 with mass flows *F*_1_ and *F*_2_ respectively. Streams 1 and 2 have compositions $c_{1}^{A}$ and $c_{2}^{A}$ of component A and $c_{1}^{B}$ and $c_{2}^{B}$ of component B. Under this conditions and assuming constant density, what is the composition in A of the stream produced by mixing streams 1 and 2? Clearly, the mass flow of the resultant stream is *F* = *F1* + *F2*. A mass balance for component A indicates that: $Fc^{A}=F_{1}c_{1}^{A}+F_{2}c_{2}^{A}$, then if *γ* = *F*_1_/*F*, we have: $c^{A}=\gamma c_{1}^{A}+\left(1-\gamma \right)c_{2}^{A}$, as in Eq. (10). Clearly, any point along a mixing line is attainable, and the duty of the mixing operations is to fill in concave regions in space. This mixing definition is intimately connected to the concepts of *convex sets* and *convex hulls*. Let us consider a subset *S* of the space of n-tuples (*S* ⊂ *R*^*n*^), we will say that *S* is convex if for every pair of points in *S*, the line connecting them is completely contained in *S*. The set shown in Figure 14 is convex, and the convex hull is the intersection of all the sets in *R*^*n*^ that contain *S*. In two dimensions it can be envisioned as the tightest rubber band that bound the set (as in Figure 14), and in higher dimensions as a convex polytope enclosed by a finite number of hyper planes.

Finally, *extreme points* are defined as points in *R*^*n*^ that lie in a vertex of the convex hull. They can neither lie in the interior of the convex hull, nor in the interior of one of the hyper planes (lines) that bound the convex hull. In Figure 14 points A and B are not extreme points since they lie in the interior of the convex hull. Point C is not extreme either because it is along one of the lines between two vertices.

Now that the necessary terminology has been introduced, we are in position to present some necessary conditions that characterize the attainable region [17], this list is not exhaustive and more properties can be founded elsewhere [20]: (i) the AR must contain the feed point, (ii) the AR must be convex, (iii) all reaction rate vectors in the boundary of the AR (*δAR*) must be tangent, point inward or be equal to 0 and (iv) no negative of a rate vector in the complement (outside) of the AR, when extended, can intersect a point of *δAR*. Since, the feed point is attainable (even without mixing or reaction) condition (i) does not require further explanation. Condition (ii) is a consequence of the fact that a set of achievable points that is not convex can always be made convex by mixing. That is, mixing can fill in concave regions or spaces between two separates, yet achievable, regions. Recall the fact that a PFR follow a trajectory that is always tangent to the rate vector; then if condition (iii) is not satisfied, a vector in the AR frontier would point outwards the AR and hence using a suitable PFR it will be possible to extend the AR. Finally, if condition (iv) is not observed; then starting from a point on the AR, a CSTR could be used to reach the point in the complement of AR where the negative rate vector originates. That is, this vector and the vector defined by the difference between the outlet and feed concentrations would be collinear, and hence a CSTR can connect both points.

### Conversion and yields definitions

The AR can be constructed in any space, as long this space obeys the mixing law defined by Eq. (10). This includes mass fractions, yields and conversions. Because they can be bounded between zero and one, and they are strictly increasing values, we choose conversions and yields as measures of the reaction extent. For any given concentration of cellulose (S, conversion *x*_*S*_), glucose (G, yield *x*_*G*_ ) and ethanol (P, yield *x*_*P*_) and its values in the feed stream denoted by a *o* subscript we have:

(11) $$\begin{array}{c} x_{S}=1-S/S_{o}\\ x_{G}=\frac{G-G_{o}}{f_{\mathrm{SG}}S_{o}}\\ x_{P}=\frac{P}{f_{\mathrm{SP}}S_{o}+f_{\mathrm{GP}}G_{o}} \end{array}$$

Where *f*_*SG*_, *f*_*SP*_ and *f*_*GP*_ are stoichiometric coefficients equal to 1.111, 0.568 and 0.511 respectively. We also consider, for the sake of simplicity, that cellobiose and ethanol are not present in any feed stream and that the conversion of every reactor in the network is based on the values in the feed stream coming from the pretreatment reactor (either washed solids or non-separated pretreatment material) as this stream represents the only feed stream of the reactors network.

### Dimensionality reduction techniques

Although it is natural to describe the dimensions of the AR in terms of the total number of species in the reaction network, this may be unnecessary because they are generally not independent. This dependence is a consequence of quantities that preserve their values during the course of a reaction. Among others, the atomic balance on the reacting species must always hold and the constraint imposed by this balance allows projecting the concentrations during the course of the reaction into a lower dimension space of independent species. That is, the constraints imposed by an invariable quantity introduce new equations that can be used to reduce the number of degrees of freedom to the extent that the remaining variables of the problem may be illustrated graphically in two or three dimensions. These projections build upon the concept of reaction invariants [30] and have been used previously to reduce the number of dimensions in which the AR must be constructed [31]. Here, we applied the same dimensionality reduction technique. Although, the method can be best explained by example, first we introduce some necessary notation. Additionally, a simpler but lengthy approach is presented in the Additional file 1.

Consider a reacting system with *i* components, being *n*_*i*_ the moles of species *i* at any time of the course of the reaction. Each component *i* is formed by *a*_*ij*_ atoms of element *j*. Let, ∆*n* be a vector of changes of the number of component moles and *A* the atom/component matrix with entries *a*_*ij*_. From the atomic balance, it follows that: *A*∆*n =* 0. Considering that ∆*n* and *A* can be partitioned as: Δ*n* = [Δ*n*_*dep*_|Δ*n*_*ind*_] and A = [*A*_*dep*_|*A*_*ind*_]. Where the sub-indices *dep* and *ind* stands for dependent and independent components. Replacing the partitioned matrices in the atomic balance, and with minor rearrangements, the dependent components change of moles can be calculated as: $\Delta n_{\mathrm{dep}}=-A_{\mathrm{dep}}^{-1}A_{\mathrm{ind}}\Delta n_{\mathrm{ind}}$. Clearly, *A*_*dep*_ has to be square and non-singular.

For the enzymatic hydrolysis reaction network, the atomic balance is given by Eq. (12) with compounds *i* = {*S*: Cellulose (*C*_6_*H*_10_*O*_5_), *G*: Glucose (*C*_6_*H*_12_*O*_6_), *B*: Cellobiose (*C*_12_*H*_22_*O*_11_), *W*: Water (*H*_2_*O*)} and atoms j = {C,H,O}

(12) $$A^{H}\Delta n^{H}=\left[\begin{array}{ccc} 6 & 6 & \begin{array}{cc} 12 & 0 \end{array}\\ 10 & 12 & \begin{array}{cc} 22 & 2 \end{array}\\ 5 & 6 & \begin{array}{cc} 11 & 1 \end{array} \end{array}\right]\left[\begin{array}{c} \Delta n_{S}\\ \Delta n_{G}\\ \Delta n_{B}\\ \Delta n_{W} \end{array}\right]=0$$

However, *A*^*H*^ clearly it is not a full rank matrix. In fact, *rank*(*A*^*H*^) = 2; that is, a row in *A*^*H*^ can be written as a linear combination of the remaining two rows (the third row can be expressed as the first row times zero plus the second row times 0.5). Hence, partitioning between independent (cellulose and glucose) and dependent components (cellobiose and water) and taking only the independent rows of *A*^*H*^, we have:

(13) $$\Delta n_{\text{dep}}^{H}=-{\left(A_{\text{dep}}^{H}\right)}^{-1}A_{\text{ind}}^{H}\Delta n_{\text{ind}}^{H}$$

(14) $$\begin{array}{c} \Delta n_{\text{dep}}^{H}=\left[\begin{array}{c} \Delta n_{B}\\ \Delta n_{W} \end{array}\right]\\ \;=-{\left[\begin{array}{cc} 12 & 0\\ 22 & 2 \end{array}\right]}^{-1}\left[\begin{array}{cc} 6 & 6\\ 10 & 12 \end{array}\right]\left[\begin{array}{c} \Delta n_{S}\\ \Delta n_{G} \end{array}\right]\\ \;=\frac{1}{2}\left[\begin{array}{c} -\Delta n_{S}-\Delta n_{G}\\ \Delta n_{S}-\Delta n_{G} \end{array}\right] \end{array}$$

This demonstrates that the change of the number of moles of water and cellobiose during the reaction course can be calculated as a function of the changes of glucose and cellulose. This also means that the AR of the enzymatic hydrolysis reaction has to be constructed in a two-dimensional space of glucose and cellulose concentration or cellulose conversion and glucose yield (and not in a four-dimensional one). Since we are interested in the residence time of the different reactor configurations, we add this variable as the third dimension of the AR. Hence, the AR of enzymatic hydrolysis must be build in the 3-dimentional space {*x*_*S*_*, x*_*G*_*, τ*}.

In the original model of ethanol fermentation, the parameters *m*_*s*_ and *m*_*p*_ in Eq. (4), have values that are close to zero so in this study these values were taken as zero. Two reasons explain this simplification. Firstly, under SSF conditions glucose concentrations reach a very low value during the reaction course. This is caused by the greater glucose demand by the biomass compared with the rate of glucose production from cellulose. Clearly, in these conditions bioethanol rate is not controlled by the glucose to ethanol rate, but by the cellulose to glucose rate. However, if the parameters *m*_*s*_ and *m*_*p*_ are not zero, then the ethanol production rate (*r*_*p*_) will be larger than the glucose production rate, which is clearly impossible. Secondly, if *m*_*p*_ and *m*_*s*_ are equal to zero, no important differences in the model predictions are observed under the conditions used in this study. In fact, if 100 g/L of glucose are taken as the initial concentration in a PFR, the only effect is a 2% increase in the residence time required for total glucose consumption and a 0.88% decrease in ethanol yield at 32°C.

Another important benefit of taking the values of *m*_*p*_ and *m*_*s*_ as zero is that the AR^c^ for glucose fermentation can be constructed in only two dimensions (ethanol yield and residence time). To understand why this is possible, note that we can calculate the reaction rates of glucose, ethanol and biomass as functions of the ethanol production rate:

(15) $$\left\{r_{x},r_{G},r_{p}\right\}=\left\{\frac{1}{Y_{P}},\frac{1}{Y_{P}Y_{x}},1\right\}r_{P}$$

This implies that glucose and biomass concentrations can be expressed as a function of ethanol concentration:

$$\begin{array}{c} X=X_{0}+\frac{\left(P-P_{0}\right)}{Y_{P}}\\ G=G_{0}-\frac{P-P_{0}}{Y_{P}Y_{x}} \end{array}$$

Finally, our ability to calculate *X* and *S* as a function of *P* allow us to also calculate the reaction rates as a function of *P* exclusively. In other words, for each value of P in the {*P, τ*} plane we can calculate a reaction vector {*r*_*p*_, 1} that uniquely determines the trajectories of the CSTR and PFR reactors from a given feed point.

Finally, to construct the AR^c^ for cSSF only three dimensions in the concentration space are required. Although a more rigorous analysis can be performed using the dimensionality reduction technique used by Omtveit et al. [31], the same results can be obtained by applying the following reasoning. If the AR^c^ for cSHF can be built in the bi-dimensional space of {*x*_*S*_*, x*_*G*_} and the AR^c^ for glucose fermentation can be reduced to only one dimension of ethanol yield, then as the two reaction networks are linked by a component present in both networks (glucose) then 3 dimensions are needed to build the AR^c^ for cSSF: {*x*_*S*_*, x*_*G*_*, x*_*P*_}. This result implies that every reaction rate in the cSSF network can be calculated from conversions and yields {*x*_*S*_*, x*_*G*_*, x*_*P*_}.

### Attainable region construction

For glucose fermentation and enzymatic saccharification (without considering the reactors residence time), AR^c^ can be constructed in two dimensions. In this space, it is possible to build the AR^c^ using the following steps [18,19]:

(i) Calculate the PFR trajectory starting from the feed point. This trajectory can be calculated by solving Eq. (7) up to a pre-established residence time.

(ii) If the PFR trajectory is not convex, find the convex hull of the PFR by drawing mixing lines to fill the non-convex parts.

(iii) Next, check along the boundary of the convex hull to see if any reaction vector points outward. If the reaction vector point outward over certain regions, then find the CSTRs that extend the region the most. If no reaction vector point outward, check if there are vectors in the complement of the AR^c^ that can be extrapolated back into the AR^c^. If this is the situation, extend the region using appropriate reactors.

(iv) Find the new, enlarged convex hull. If a CSTR lies in the boundary, the reaction vector at this point must point out of the AR^c^, and a PFR with feed point on the CSTR will extend the region.

(v) Repeat steps (iii) and (iv), alternating between PFRs and CSTRs until no reaction vectors point out over the AR^c^, and the necessary conditions are met.

As was stated by Glasser and Hildebrandt [17], this constructive procedure implies that for a two dimensional system, the boundary of the attainable region “*must be achieved by a sequential process and must consist of alternate straight lines and plug-flow trajectories*”.

For cSSF and cSHF (considering the residence time), the AR^c^ must be built in a three dimensional space. For cSSF, we choose cellulose conversion, glucose and ethanol yields as these dimensions since they provide useful insights regarding: the liquefaction process, as this process depends on cellulose conversion; the yield and productivity of the product of interest, related to ethanol conversion and the glucose yield since glucose is the compound that links the enzymatic hydrolysis and fermentation processes.

The construction of a three dimensional AR^c^ is far more difficult than the previously described process for two dimensions. Regardless of these difficulties, powerful theoretical results were derived in a series of papers [20-22]. These theoretical results were recently used to formulate an automated algorithm for AR^c^ construction [32] and we follow this algorithm to analyze the cSSF and cSHF reaction networks and build the candidate attainable regions. The algorithm can be summarized in the following steps:

• Calculate the PFR and CSTR trajectories from the feed point. Stop the calculations when the maximum user defined value of residence time is achieved. Calculate the convex hull formed by these trajectories.

• Create a set of constant feed rate (*α*) values such that *α* = [0, *α*_1_, *α*_2_, …, *α*_*large*_]. Calculate the DSR trajectories (Eq. (9)) for each *α* value from each available extreme point (such as feed point and equilibrium points). Then calculate the convex hull of these trajectories, eliminate the interior points and store the extreme points. These extreme points lie on the extreme DSR as defined by Feinberg [21].

• If necessary, refine the set of *α* values to produce more points in the extreme DSR trajectory. A stopping criterion suitable for automation of the algorithm is given elsewhere [32], however we refined the set of *α* values manually.

• From each extreme point on the DSR extreme trajectory, generate PFRs with feed points along these points. Calculate the convex hull of the enlarged region created by these trajectories.

We verified our ability to apply the above described methodology by reproducing the results of Example 1: 3D Van de Vusse type kinetics in Seodigeng et al. [32].

### Software and computational tools

MATLAB® was used to perform all calculations in this work. To solve systems of ordinary differential equations (ODE), such as the ODEs that define the PFR and DSR trajectories, we used the MATLAB built-in ODE45 algorithm based on explicit Runge–Kutta formula. Systems of algebraic equations, defining CSTR trajectories, were solved using fmincon solver and its built-in interior point method [33]. For convex hull calculation, the MATLAB convhull solver was used. This tool is based on the Qhull algorithm developed by Barber et al. [34].

## Abbreviations

ARc: Candidate attainable region; cSSF: Continuous simultaneous saccharification and fermentation; cSHF: Continuous separated hydrolysis and fermentation; DW: Dry weight; nSPM: Non-separated pretreated material; RNS: Reactor network synthesis.

## Competing interests

The authors declare no financial or non-financial competing interests.

## Authors’ contributions

FS, GA, and RC conceived of and participated in the design of the study. FS performed the calculations and wrote a draft of this work. RC and GA provided oversight during the calculations and development of the first draft of the manuscript. FS, GA, and RC edited several versions of the manuscript. All authors read and approved the final manuscript.

## Supplementary Material

Additional file 1 Reaction invariants and algorithmic construction of the attainable region. Click here for file
