# Supplementary material for: Attainable region analysis for continuous production of second generation bioethanol
Source: Biotechnol Biofuels. 2013 Nov 29;6:171. doi: 10.1186/1754-6834-6-171 (PMC3879065; doi:10.1186/1754-6834-6-171)
Supplement: Additional file 1 — Reaction invariants and algorithmic construction of the attainable region. [file 1754-6834-6-171-S1.pdf]

Supporting Information Material for

Attainable region analysis for continuous production of second generation bioethanol

Supplementary material S1

Reaction invariants and the reduction of the AR dimension

The article that motivates this supplementary material, gives a more concise treatment of the used dimensionality reduction techniques, in this document they are presented in a more intuitive manner. Reaction invariants are quantities that preserve their values during a chemical or biochemical reaction. Although the number of moles of each species changes in a reaction, the constituent atoms of these species must be conserved.

$$(C_6H_{10}O_5)_n + H_2O \overset{r_1}{\rightarrow} C_{12}H_{22}O_{11} + (C_6H_{10}O_5)_{n-2} \quad \text{(S1)}$$

$$C_{12}H_{22}O_{11} + H_2O \overset{r_2}{\rightarrow} 2C_6H_{12}O_6 \quad \text{(S2)}$$

$$(C_6H_{10}O_5)_n + H_2O \overset{r_3}{\rightarrow} C_6H_{12}O_6 + (C_6H_{10}O_5)_{n-1} \quad \text{(S3)}$$

Hence, we may formulate atom balances for any reaction network. For enzymatic saccharification, the model reaction network shown in Eqs. S1 to S3, comprises four species: cellulose, $(C_6H_{10}O_5)_n$; glucose, $C_6H_{12}O_6$; cellobiose, $C_{12}H_{22}O_{11}$; and water, H_2O. Let be n_S, n_G, n_B and n_W the number of moles of cellulose, glucose, cellobiose and water at any extent (or time) of the reaction. Since the total number of C, H or O atoms in the system cannot change, irrespective of the extent of the reactions, we can formulate three linear balance equations connecting the four species.

Carbon balance: $6n_S + 6n_G + 12n_B + 0n_W = constant$

Hydrogen balance: $10n_S + 12n_G + 22n_B + 2n_W = constant$

Oxygen balance: $5n_S + 6n_G + 11n_B + 1n_W = constant$

If instead of the total number of moles of each species, the linear balances are written in terms of the change in the number of moles of each species, then the linear balances can be equated to zero.

Carbon balance: $6\delta n_S + 6\delta n_G + 12\delta n_B + 0\delta n_W = 0$

Hydrogen balance: $10\delta n_S + 12\delta n_G + 22\delta n_B + 2\delta n_W = 0$

Oxygen balance: $5\delta n_S + 6\delta n_G + 11\delta n_B + 1\delta n_W = 0$

Note that the hydrogen and oxygen balances are not linearly independent; hence the system of equations comprises four unknowns and two linearly independent equations. This means that two dependent variables can be expressed as a function of the remaining two independent variables. For our purpose is convenient to choose the variations in the numbers of moles of cellulose and glucose as the independent variables, hence solving the system of equations comprised of the C and H balances to calculate δn_B and δn_W we have:

$$\delta n_B = -\frac{\delta n_S + \delta n_G}{2}$$

$$\delta n_W = \frac{\delta n_S - \delta n_G}{2}$$

Since, using these expressions, we can calculate the variations in the number of moles of water and cellobiose as a function of the changes of glucose and cellobiose, there is no need of representing the AR in a four-dimensional space, but only in a two-dimensional. One may therefore, use the attainable region concept in two dimensions since only two of the four variables in the problem are linearly independent.

Algorithmic construction of the AR^c for cSSF

In this section, the construction of the AR^c for cSSF is presented in an algorithmic fashion. The procedure was taken from the work of Seodigeng *et al.* [1]. Although, the steps in the construction of the AR^c were already given in the article that motivates this supplementary material, they are reproduced here for the reader's convenience:

i. Calculate the PFR and CSTR trajectories from the feed point. Stop the calculations when the maximum user defined value of residence time is achieved. Calculate the convex hull formed by these trajectories.
ii. Create a set of constant feed rate (α) values such that $\alpha = [0, \alpha_1, \alpha_2, \ldots, \alpha_{large}]$. Calculate the DSR trajectories for each α value from each available extreme point (such as feed point and equilibrium points). Then calculate the convex hull of these trajectories, eliminate the interior points and store the extreme points. These extreme points lie on the extreme DSR as defined by Feinberg [2].
iii. If necessary, refine the set of α values to produce more points in the extreme DSR trajectory. A stopping criterion suitable for automation of the algorithm is given elsewhere [1], however we refined the set of α values manually.
iv. From each extreme point on the DSR extreme trajectory, generate PFR with feed points along these points. Calculate the convex hull of the enlarged region created by these trajectories.

The equations required to apply the algorithm S1 are summarized in the systems of equations PFR-Tr, CSTR-Tr and DSR-Tr. The equations required to calculate the conversions and yields of each species using mass concentrations are detailed in Eq. (S4).

The kinetic expression r_1, r_2, r_3, r_G^F, r_x^F and r_P^F are detailed in the article, or alternatively, references to articles from which these expressions were taken are provided.

PFR trajectory

$$\frac{d}{d\tau}\begin{bmatrix} c_s(\tau) \\ c_B(\tau) \\ c_G(\tau) \\ c_x(\tau) \\ c_P(\tau) \end{bmatrix} = \begin{bmatrix} -r_1 - r_2 \\ r_1 - r_2 \\ r_2 + r_3 - r_G^F \\ r_x^F \\ r_P^F \end{bmatrix}, \qquad \boldsymbol{c}(\tau = \tau^0) = \boldsymbol{c^0} \tag{PFR-Tr}$$

CSTR trajectory

$$\begin{bmatrix} c_S^0 - c_s(\tau) \\ c_B^0 - c_B(\tau) \\ c_G^0 - c_G(\tau) \\ c_x^0 - c_x(\tau) \\ c_P^0 - c_P(\tau) \end{bmatrix} = -\tau \begin{bmatrix} -r_1 - r_2 \\ r_1 - r_2 \\ r_2 + r_3 - r_G^F \\ r_x^F \\ r_P^F \end{bmatrix} \tag{DSR-Tr}$$

DSR trajectory

$$\frac{d}{d\tau}\begin{bmatrix} c_s(\tau) \\ c_B(\tau) \\ c_G(\tau) \\ c_x(\tau) \\ c_P(\tau) \end{bmatrix} = \begin{bmatrix} -r_1 - r_2 \\ r_1 - r_2 \\ r_2 + r_3 - r_G^F \\ r_x^F \\ r_P^F \end{bmatrix} + \alpha \begin{bmatrix} c_S^F - c_s(\tau) \\ c_B^F - c_B(\tau) \\ c_G^F - c_G(\tau) \\ c_x^F - c_x(\tau) \\ c_P^F - c_P(\tau) \end{bmatrix}, \qquad \boldsymbol{c}(\tau = \tau^0) = \boldsymbol{c^0} \tag{DSR-Tr}$$

Conversions and yields definitions

$$x_S(\tau) = 1 - c_s(\tau)/c_S^0$$

$$x_G(\tau) = \frac{c_G(\tau) - c_G^0}{c_S^0 f_{SG}} \quad \text{(S4)}$$

$$x_P(\tau) = \frac{c_P(\tau)}{f_{SP}c_S^0 + f_{GP}c_G^0}$$

Algorithm S1

Step (i)

Calculate the PFR trajectory from F

- Calculate the PFR trajectory $c_{PFR}(\tau) = \{x_S(\tau), x_G(\tau), x_P(\tau)\}$ by solving the system of differential equations PFR-Tr with $\boldsymbol{c}^0 = F$ and $\tau^0 = 0$.
- Store the residence time values: $\tau_{PFR} = \tau$

Calculate the CSTR trajectory from F

- Calculate the CSTR locus $c_{CSTR}(\tau) = \{x_S(\tau), x_G(\tau), x_P(\tau)\}$ by solving the system of differential equations CSTR-Tr with $\boldsymbol{c}^0 = F$ and $\tau^0 = 0$.
- Store the residence time values: $\tau_{CSTR} = \tau$

Calculate the PFR trajectories with feed points along the CSTR locus

- Select J values of residence time between zero and the maximum value of τ_{CSTR}, the index of this set is j such that $\tau_{CSTR,i}$ is an element of τ_{CSTR}.
- Calculate the CSTR→PFR trajectories: $c_3(\tau)_i = \{x_S(\tau), x_G(\tau), x_P(\tau)\}$ by solving J systems of differential equations PFR-Tr with $\boldsymbol{c}^0 = c_{CSTR}(\tau_{CSTR,i})$ and $\tau^0 = \tau_{CSTR,i}$
- Store the residence time values: $\tau_3 = \tau$

Calculate the convex hull and store the extreme points

- Append the trajectories calculated so far: $\boldsymbol{C}(\tau) = [c_{PFR}(\tau), c_{CSTR}(\tau), c_3(\tau)_i]$
- Calculate the convex hull of the region and store the extreme points: $\boldsymbol{C}^* = convhull(\boldsymbol{C}(\tau))$

Step (ii)

Calculate DSRs with feed points along $\boldsymbol{C}^*$

- Side feed rate values such that $\boldsymbol{\alpha} = [0, \alpha_1, \alpha_2, \ldots, \alpha_{large}]$, 50 points exponentially spaced.

- For all point in $\boldsymbol{C}^*$ and for all point in $\boldsymbol{\alpha}$ calculate the DSR trajectories $c_{DSR,k}(\tau)$ solving the system of differential equations DSR -Tr with $\boldsymbol{c}^0 = \boldsymbol{C}^*{}_k$, where $\boldsymbol{C}^*{}_k$ are each one of the elements in $\boldsymbol{C}^*$ and $\boldsymbol{c}^F = F$.

Calculate the convex hull and store the extreme points

- Append the trajectories calculated so far: $\boldsymbol{C}(\tau) = [all\ c_{DSR}(\tau)\ ]$
- Calculate the convex hull of the region and store the extreme points: $\boldsymbol{C}^* = convhull(\boldsymbol{C}(\tau))$

Step (iii)

It was not necessary to refine the side feed rate partition

Step (iv)

Calculate the PFR trajectories with feed points along the DSR extreme points $\boldsymbol{C}^*$

- Calculate PFR trajectories: $c_4(\tau)_l = \{x_S(\tau), x_G(\tau), x_P(\tau)\}$ by solving as many systems of differential equations PFR-Tr as points in $\boldsymbol{C}^*$ with $\boldsymbol{c}^0 = \boldsymbol{C}^*{}_l$ and $\tau^0 = \tau_{DSR,i}$ where $\boldsymbol{C}^*{}_l$ are each one of the elements in $\boldsymbol{C}^*$ and $\tau_{DSR,i}$ their corresponding residence time.
- Store the residence time values: $\tau_{4,l} = \tau_l$

Calculate the convex hull and store the extreme points

- Append the trajectories calculated so far: $\boldsymbol{C}(\tau) = [all\ c_4(\tau)_l\ , \boldsymbol{C}^*{}_2, \boldsymbol{C}^*{}_1]$
- Calculate the convex hull of the region and store the extreme points: $\boldsymbol{C}^*{}_3 = convhull(\boldsymbol{C}(\tau))$

References

1. Seodigeng T, Hausberger B, Hildebrandt D, Glasser D: **Recursive constant control policy algorithm for attainable regions analysis**. *Computers & Chemical Engineering* 2009, **33**:309–320.

2. Feinberg M: **Optimal reactor design from a geometric viewpoint. Part II. Critical sidestream reactors**. *Chemical Engineering Science* 2000, **55**:2455–2479.
